# Construction of ceRNA networks of lncRNA and miRNA associated with intramuscular fat deposition in Ujumqin sheep

**DOI:** 10.3389/fvets.2025.1559727

**Published:** 2025-03-19

**Authors:** Mingxi Lan, Qing Qin, Yuchun Xie, Chongyan Zhang, Zhichen Liu, Xiaolong Xu, Jingwen Zhang, Songsong Xu, Ji Yang, Haijun Zhang, Suhe Alatan, Zhixin Wang, Zhihong Liu

**Affiliations:** ^1^Animal Science Department, Inner Mongolia Agricultural University, Hohhot, China; ^2^College of Animal Science and Technology, Hebei Science and Technology Normal University, Qinhuangdao, Hebei, China; ^3^Key Laboratory of Sheep & Goat Genetics, Breeding and Reproduction in Inner Mongolia, Inner Mongolia Agricultural University, Hohhot, China; ^4^Key Laboratory of Sheep & Goat Genetics and Breeding of Ministry of Agriculture Inner Mongolia Agricultural University, Hohhot, China; ^5^College of Animal Science and Technology, China Agricultural University, Beijing, China; ^6^Erdos Agricultural and Animal Husbandry Science Research Institute, Ordos, China; ^7^East Ujumqin Banner Hishig Animal Husbandry Development Co., Ltd., East Ujumqin Banner, China

**Keywords:** sheep, IMF, lncRNA, miRNA, ceRNA network

## Abstract

**Introduction:**

The molecular mechanisms underlying intramuscular fat (IMF) deposition are crucial for enhancing lamb meat quality. This process is regulated by a network of transcription factors. Exploring the role of non-coding RNAs, particularly lncRNAs and miRNAs, in IMF deposition can clarify its complex genetics and offer resources for breeding Inner Mongolian local breeds.

**Methods:**

We evaluated carcass and lamb meat quality parameters using 60 six-month-old Ujumqin sheep with similar body weights. To investigate non-coding RNA’s role in IMF deposition, we identified differentially expressed genes and pathways between the longissimus dorsi and femoral biceps. Additionally, we analyzed these genes and the lncRNA-miRNA-mRNA co-regulatory network in high- and low-IMF femoral biceps groups.

**Results:**

We identified 11,529 mRNAs (747 differentially expressed), 9,874 lncRNAs (1,428 differentially expressed), and 761 miRNAs (12 differentially expressed). GO and KEGG enrichment analyses showed these genes are involved in lipid metabolism, fatty acid oxidation, and energy metabolism. We constructed a ceRNA network with 12 lncRNAs, 4 miRNAs, and 6 mRNAs. Notably, lncRNA MSTRG.13155.1 interacts with miR-1343-3p_R + 2, promoting IMF deposition by releasing HADHA gene expression. Dual-luciferase reporter assays confirmed MSTRG.13155.1 and HADHA as miR-1343-3p_R + 2 targets. RT-qPCR validated the expression trends of key mRNAs, miRNAs, and lncRNAs, consistent with sequencing results.

**Discussion:**

Our comprehensive analysis of differentially expressed genes and pathways in Ujumqin sheep’s longissimus dorsi and femoral biceps, along with high- and low-IMF groups, has revealed the complex genetics of IMF deposition. This offers valuable resources for Inner Mongolian local breed selection. The interaction between lncRNA MSTRG.13155.1 and miR-1343-3p_R + 2, and their regulation of HADHA expression, provides new insights into IMF deposition mechanisms. Future research can explore these mechanisms’ universality and specificity across different breeds and environments.

## 1 Introduction

The Ujumqin sheep, a prized local breed in Inner Mongolia, is celebrated for its remarkable disease resistance, adaptability to natural grazing, and tolerance to rough feeding conditions. Characterized by rapid growth, high meat yield, tender meat, and efficient fat deposition, this breed has become a cornerstone of the region’s sheep meat industry. Sheep carcass adipose tissue is primarily composed of three components, namely: intermuscular fat, intramuscular fat (IMF), and subcutaneous fat. Although IMF constitutes only 1 to 2% of total carcass lipids, its role in enhancing meat palatability and flavor is pivotal (1, 2). The presence of IMF creates a marbling effect within the muscle, altering the cross-linking structure between muscle fiber bundles and presenting a homogeneous reticulation. This structural modification provides spaces for water accumulation, thereby increasing meat tenderness and juiciness (3). The development of IMF begins during the late embryonic stage through the differentiation of a select subset of skeletal muscle cells. As these adipocytes mature, lipid droplets appear within the cytoplasm and gradually expand to fill the entire cell, establishing sites for IMF accumulation. The quantity and size of these intramuscular adipocytes ultimately determine the IMF content to a large extent (4, 5). However, IMF deposition varies significantly across different muscle groups. The study conducted by Li et al. (6) examined the meat quality parameters of various Sunit sheep muscles and found that the longest dorsal muscle exhibited superior tenderness and water retention compared to the biceps femoris muscle. Conversely, the biceps femoris muscle had higher IMF content, lower drip loss, smaller myofiber diameters, and richer fatty acid compositions. Studies also observed histological differences in the longest dorsal muscle of small-tailed frigid sheep, noting that the ADIPOQ gene was negatively correlated with IMF content, while the PPARGC1A gene showed a positive correlation (7). Despite these advancements, few studies have explored IMF deposition specifically in the biceps femoris muscle. Selective fat deposition, which can enhance productivity and significantly improve meat quality, remains a critical challenge in livestock breeding (8). As an important branch of Mongolian sheep, the Ujumqin sheep was among the first breeds recognized for lamb fattening in China (9), contributing significantly to Inner Mongolia’s sheep meat production. Therefore, identifying the key effector genes involved in IMF deposition across different muscle regions and elucidating the mechanisms governing IMF accumulation in meat sheep is essential for improving lamb meat quality.

IMF deposition is a complex process regulated by multiple transcription factors and their networks, including mRNAs, long noncoding RNAs (lncRNAs) and microRNAs (miRNAs). The key transcription factors that have been well studied are peroxisome proliferator-activated receptor gamma (PPAR-*γ*) (10) and the CCAAT enhancer binding protein family (CCAAT). CCAAT enhancer binding protein family, CEBPs (11), fatty acid binding protein 3 (FABP3) (12), lipoprotein lipase (LPL) (13). Glycerolipid metabolism and fatty acid degradation pathways also contribute to differences in IMF deposition (14). Each of these candidate genes and pathways have also been used as molecular marker potential for meat quality trait selection. Studies on lncRNAs regulate preadipocyte differentiation or adipogenesis by demonstrating competitive mechanisms with endogenous RNAs (ceRNAs). For example, lncRNA IRLNC (15) affects IMF catabolism by regulating the expression of nuclear receptor subfamily 4, clade A member 3, and lnc-BATE1 (16) was found to regulate brown adipocyte growth and thermogenesis in transcriptomic data from different adipose tissues in mice. There are also several related studies reporting that lncRNAs play a key regulatory role in sheep tail fat deposition (17) and are involved in the regulation of adipocyte growth. miRNAs are short 19-23 nt RNAs which are transcribed from endogenous genomes and distributed throughout the cell (18). In recent years, many researchers have explored about miRNAs exerting functions in animal adipose tissues through high-throughput techniques, and there have been a large number of reports about miRNAs affecting fat deposition in animals. For example, during porcine adipogenesis, miR-17-5p (19) and miR-27 (20) were both associated with the expression of fatty acid binding protein 4 (FABP4) and PPAR-*γ* and inhibited the differentiation of precursor adipocytes, while miR-196a (21) and miR-32-5p (22) promote precursor adipocyte differentiation. mRNAs can function as target genes for both lncRNAs and miRNAs, and lncRNAs can also regulate miRNAs. However, relatively few studies have been conducted to jointly analyze the effects of lncRNAs, miRNAs, and mRNAs on IMF deposition.

In sheep farming, the content of IMF is crucial for producing high-quality lamb meat. This study employed Ujumqin sheep as the research model, screening for high and low IMF groups in two specific muscles—the longest dorsal muscle (LD) and the biceps femoris (BF)—at 6 months of age. Utilizing RNA-Seq technology, we systematically identified differentially expressed genes and pathways between these two muscles. Building on this foundation, we conducted an in-depth analysis of the differentially expressed genes within the high and low IMF groups of the BF muscle and explored the lncRNA-miRNA-mRNA co-regulatory network. This comprehensive approach aims to elucidate the intricate genetic structure associated with IMF in Ujumqin sheep, providing valuable resources and a theoretical basis for the selection and breeding of local fine breeds in Inner Mongolia.

## 2 Materials and methods

### 2.1 Animals and sample collection

The samples were collected in accordance with the guidelines for laboratory animals of the Ministry of Science and Technology of China (Beijing, China) and approved by Inner Mongolia Agricultural University (approval number: (2020) 056). All samples were collected in accordance with the International Animal Research Guidelines. Sixty samples were collected from 6-month-old Ujumqin sheep from East Ujumqin Banner, Inner Mongolia, and muscle samples from LD and BF of the dorsal region were selected after slaughter, and 5 g of the samples were put into enzyme-free freezing tubes in a dispenser bag (the rest of the samples were preserved in dry ice and brought back to the laboratory for spare parts). The sample is promptly immersed in liquid nitrogen, transported to the laboratory, and subsequently stored at –80 °C in a freezer for future use.

Determination of carcass traits: Meat goats were fasted for 24 h before slaughter, and a quiet environment and sufficient water were maintained. According to the local standard Technical Procedures for Meat Sheep Performance Determination (DB42/T 1618-2021), 9 carcass traits were determined, including live weight before slaughter, carcass weight, eye muscle area, GR value, loin muscle thickness, thigh muscle thickness and slaughter rate. Meat quality determination of mutton: IMF, protein content, shear force, tethering force, 45 min and PH value (PH-Star) and 45 min meat colour (L*, a*, b*) were determined according to T/CAAA 102-2023 ‘Technical Specification for Measurement of Mutton Meat Quality’ issued by China Animal Husbandry Association. Technical Specification for Meat Quality Determination of Mutton. Free fatty acids were determined by the hydrolysis-extraction method according to the national standard GB 5009.168-2016 ‘Determination of fatty acids in foodstuffs’.

**Figure 1:**
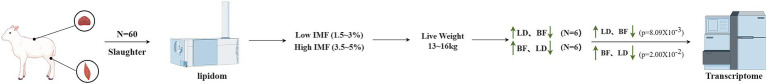
Sample selection diagram. We are incredibly grateful to East Ujumqin Banner Hishig Animal Husbandry Development Co. for data support and figdraw software for the technical support.

Whole transcriptome sequencing sample selection: Whole transcriptome sequencing samples were selected based on the IMF content and lipidomics of the longest dorsal muscle and biceps femoris muscle samples of all 60 Ujumqin sheep. Based on the selection criteria outlined in Figure 1, an initial IMF content range of 1.5 to 5% was selected. Specifically, IMF contents between 1.5–3% were categorized as the low IMF group, while IMF contents between 3.5–5% were classified as the high IMF group. From these categories, 20 samples with comparable carcass weights (ranging from 13 to 16 kg) were selected for further analysis, while 6 samples with higher IMF in LD than in BF (statistically significant difference between groups *p =* 8.09 × 10^−3^) and 6 samples with higher IMF in BF than in LD were selected (statistically significant difference between groups *p* = 2.00 × 10^−2^). The difference was statistically significant, a total of 24 samples (including 12 dorsal longest muscle and 12 biceps femoris) were subjected to whole transcriptome sequencing.

### 2.2 RNA extraction and library preparation

Total RNA was extracted by the Trizol method, and the total RNA quantity and purity were analyzed using a Bioanalyzer 2,100 and an RNA 6000 Nano LabChip Kit, with an RIN value >7.0. The ribosomal RNA (rRNA) was depleted to construct the first-strand cDNA library. After the library passed the quality inspection, Illumina NovaSeqTM 6,000 (LC-Bio Technology Co., Ltd., Hangzhou, China) was used for sequencing, and the sequencing read length was 2 * 150 bp (PE150).

### 2.3 Raw data quality control

In order to obtain high-quality clean reads through Cutadapt (https://cutadapt.readthedocs.io/en/stable/, version: Cutadapt 1.9), to further filter the reads. The parameters are as follows: reads containing adapters were removed; reads containing polyA and polyG were removed; reads with more than 5% unknown nucleotides (N) were removed; and reads with more than 20% low-quality bases (*q* value ≤20) were removed. Then, FastQC^1^ was employed to verify sequence quality, which includes Q20. Q30, and the GC content of the clean data.

### 2.4 Identification and screening of RNA-seq

First, transcripts that overlapped with known mRNAs and lncRNAs were screened out. Then, the CPC 0.9-r2^2^ and CNCI 2.0^3^ were used with their default parameters to predict new transcripts with coding potential. Finally, we retained transcripts with a Coding Potential Calculator (CPC) score less than 0.5 and a Coding-Non-Coding Classifier (CNCI) score less than 0, designating them as novel lncRNAs.

### 2.5 Differential expression gene screening

DESeq2 software was used to analyze the differential expression of genes between the two groups. The screening criteria for lncRNA and mRNA differences were as follows: transcripts with an error rate of FDR < 0.05 and |Fold Change| ≥ 1.5 were considered to be differentially expressed. For miRNA identification, FastQC was used for quality control with its default parameters.

### 2.6 Target gene prediction and functional analysis

lncRNA uses a Python script to screen the upstream and downstream 100,000 bp coding genes as the cis-acting potential target genes of lncRNA and analyze their functions. The GO (Gene Ontology) seq package in the R language is used for GO enrichment analysis, and KOBAS software is used for KEGG (Kyoto Encyclopedia of Genes and Genomes) pathway enrichment analysis.

The lncRNA and 3´UTR sequences of mRNA were predicted as miRNA targets using TargetScan (5.0) and miranda (3.3a). When TargetScan_score is ≥50 (the TargetScan threshold) and miranda_Energy is < −10 (the miranda threshold), the intersection of the two software predictions is taken. TargetScan is based on the seed region prediction of miRNA targets, while miranda is mainly based on the free energy between lncRNA, mRNA, and miRNA. The lower the free energy, the stronger the bond between the two. Therefore, TargetScan and miranda were selected to predict the targeting relationship between lncRNA, mRNA, and miRNA.

### 2.7 Construction of lncRNA-miRNA-mRNA network

The online website miRDB^4^ was used to predict the targeting relationship between miRNAs, lncRNAs, and mRNAs through sequence complementation. Combined with the target gene information, the lncRNA-miRNA-mRNA interaction network was constructed using Cytoscape software. This approach facilitates a better understanding of the regulatory relationship between the three.

### 2.8 Double luciferase reporter gene assay

The plasmid and miRNA were transfected when the cell density reached more than 70% overnight. 48 h after transfection, the Luciferase^®^ Reporter Assay System (Promega E1901) was used to determine luciferase activity.

### 2.9 RT-qPCR detection of key mRNA\lincRNA\microRNA detection

To verify the accuracy of the sequencing data, real-time fluorescent quantitative polymerase chain reaction (RT-qPCR) technology was used to select 3 lncRNAs and 3 mRNAs that were differentially expressed during fat deposition in the DE lncRNA database for real-time fluorescence quantitative PCR. GAPDH was selected as the internal reference for lncRNAs. Real-time fluorescence quantification was performed with TBGreen^®^ Premix ex TaqTM Ι (TII RNaseH PIUS) (2X) (Shanghai BioEngineering Technology Co., LTD.) in a 20 μL system: 2 × TB Green^®^ Premix ex TaqTM Ι 10 μL, upstream and downstream primers (10 μmol/μL) 0.8 μL, ROX Reference Dye (50X) 0.4 μL, cDNA (500 nm/μL) 2 μL, ddH_2_O 6 μL. The reaction procedure was as follows: pre-denaturation at 95°C for 10 min; denaturation at 95°C for 2 s, annealing at the optimum temperature for 20 s, extension at 70°C for 10 s, 40 cycles; finally, 95°C for 15 s, 60°C for 1 min, 95°C for 1 s, and storage at 4°C.

The miRNA primers were designed using the stem-loop method, and U6 was selected as the relative quantitative internal reference. The synthesis was commissioned by Nanjing Nuoweizen Biotechnology Co., LTD. The reaction system was 20 μL: 2 × miRNA Universal SYBR qPCR Master Mix 10 μL, Specific Primer (10 μM) 0.4 μL, mQ Primer R (10 μM) 0.4 μL, miRNA first-strand cDNA (500 nm/μL) 2 μL, ddH_2_O 7.2 μL. The reaction conditions were as follows: denaturation at 95°C for 5 min, denaturation at 95°C for 10 s, annealing and extension at 60°C for 30 s, 40 cycles; Finally, 95°C for 15 s, 60°C for 1 min, 95°C for 1 s, and 4°C were stored. The relative expression of miRNA was calculated by 2^−ΔΔCT^.

### 2.10 Statistical analysis

SPSS 21.0 software was used to analyze the significance of single factor ANOVA and independent sample t test. Results the mean value ± standard error was used, and the difference was significant at *p* < 0.05. *p* < 0.01 indicates a significant difference. The images of real-time fluorescence quantitative results were plotted by GraphPad Prism V9.5 software.

## 3 Result

### 3.1 Phenotypic determination of meat quality and carcass traits in Ujumqin sheep

**Figure 2:**
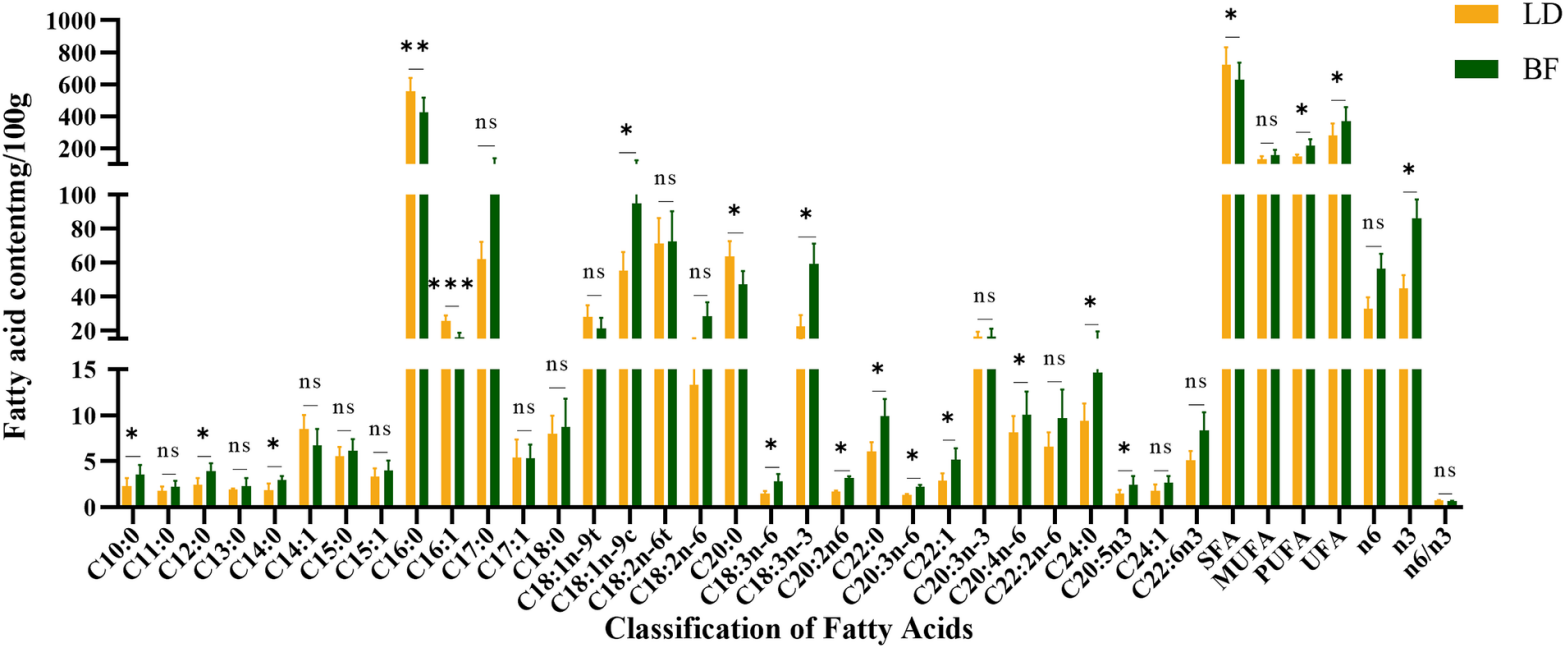
LD and BF fatty acid content detection (^**^p < 0.001, ^*^p < 0.05 compared with the LD group).

We selected 60 Ujumqin sheep that were 6-months-old and had similar body weights for the experiment, and nine slaughter traits and meat traits of these sheep were determined (Tables 1, 2). The fatty acid content and the differences between different parts are shown in Figure 2. Based on the results of IMF content and fatty acid comparison, we screened 12 samples from each group for further study (including 12 from LD part and 12 from BF part, totaling 24 samples).

**Table 1:** Statistical table of slaughter characters of 60 Ujumqin sheep.

Index	*N*	Min	Max	$\bar{x}$ ± SD
Live weight before slaughter/kg	60	27.00	40.60	33.84 ± 3.22
Carcass weight/kg	60	11.38	19.25	14.69 ± 1.77
Carcass diagonal length/cm	60	58.00	68.00	63.57 ± 2.28
Carcass straight length/cm	60	56.00	66.00	61.05 ± 2.05
Eye muscle area/cm^2^	60	9.10	19.53	13.55 ± 2.76
GR value/cm	60	0.60	0.90	0.78 ± 0.10
Waist muscle thickness/cm	60	1.20	1.86	1.57 ± 0.16
Thigh muscle thickness/cm	60	3.00	4.20	3.79 ± 0.27
Slaughter rate/%	60	39.66	49.84	45.17 ± 2.64

**Table 2:** Statistical table of meat quality traits of LD and BF of 60 Ujumqin sheep.

Index	*N*	LD	BF	*P*
IMF/%	60	2.85 ± 0.65	2.84 ± 0.62	0.190
Protein content/%	60	20 ± 0.78	19.66 ± 0.61	0.018
Shear force/*N*	60	55.57 ± 12.57	61.14 ± 8.88	0.004
Water loss rate/%	60	5.18 ± 0.19	3.77 ± 0.15	0.001
PH45min	60	6.68 ± 0.35	6.66 ± 0.20	0.972
PH24h	60	5.91 ± 0.33	5.79 ± 0.03	0.418
L*	60	28.86 ± 4.34	28.73 ± 4.27	0.811
a*	60	23.93 ± 5.87	26.11 ± 7.45	0.038
b*	60	4.29 ± 2.38	3.34 ± 2.65	0.039

As shown in Table 2, there were significant differences in protein content, shear force, water loss and meat color values (L*, a*, b*) between LD and BF parts. As shown in Figure 2, a total of 31 fatty acids were detected in Ujumqin sheep, in which the relative content of saturated fat counts was significantly higher in the LD group than in the BF group (*p <* 0.05), but the absence of unsaturated fatty acids was significantly higher in the BF group than in the LD group (*p <* 0.05). Among the saturated fatty acids, there were significant differences (*p <* 0.05) in decanoic acid (C10:0), lauric acid (C12:0), myristic acid (C14:0), palmitic acid (C16:0), arachidic acid (C20:0), behenic acid (C22:0), and methyl lignocellulosic acid (C24:0) in the LD group and the BF group. In the unsaturated fatty acid group BF group was significantly higher (*p <* 0.05) than LD group, especially the beneficial polyunsaturated fatty acids *γ*-linolenic acid (C18:3n-6), (*α*-linolenic acid C18:3n-3), and arachidonic acid (C20:4n-6).

These results indicate that the LD and BF site samples we collected are accurate and reliable for whole transcriptome sequencing analysis. The differences in fatty acid composition and meat quality traits between the LD and BF parts provide important insights into the molecular mechanisms underlying IMF deposition and meat quality formation in Ujumqin sheep. Further studies on the gene expression profiles and regulatory networks in these two muscle parts will help to identify key genes and pathways involved in IMF deposition and meat quality improvement.

### 3.2 Identification of differentially expressed genes (DEG) in LD and BF

In this study, two libraries were constructed: one for lncRNAs and mRNAs, and another for miRNAs (Supplementary Tables S1, S2). Descriptive statistics summarizing the transcriptome data indicated that the quality of the data was exceptionally high, with each sample yielding over 10 gigabases of valid data, a GC content exceeding 40%, and Q20 and Q30 values surpassing 95%.

**Figure 3:**
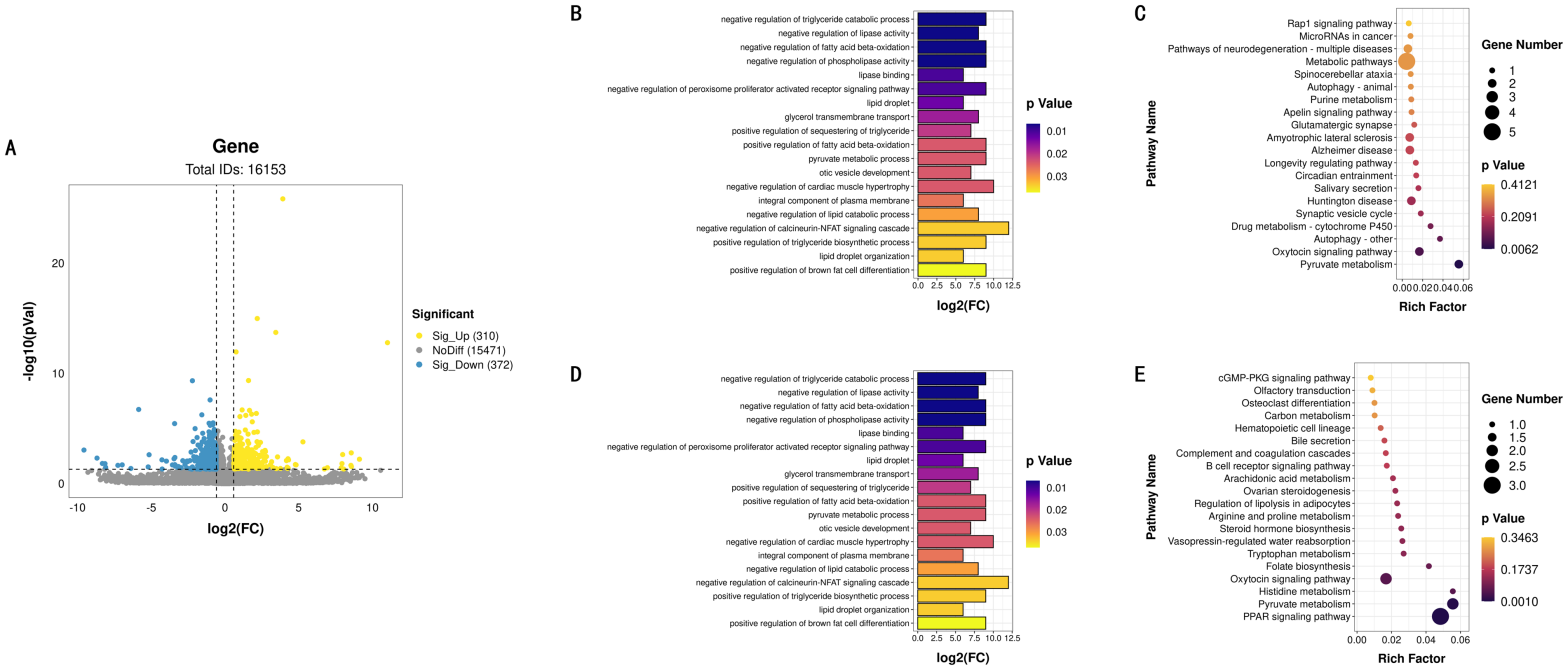
LD and BF differential expression identification. **(A)** LD and BF differential gene volcano map. **(B)** GO functional enrichment histogram of up-regulated genes. **(C)** KEGG function rich distribution map of up-regulated genes. **(D)** GO functional enrichment histogram of down-regulated genes. **(E)** KEGG function rich distribution map of down-regulated genes.

To investigate gene expression differences between tissues, we selected 12 samples from LD and 12 samples from BF. Using DESeq2 for analysis, a total of 682 differentially expressed genes (DEGs) were identified between LD and BF (Figure 3A), comprising 310 up-regulated and 372 down-regulated genes. To further elucidate the functions of these DEGs, GO and KEGG enrichment analyses were conducted separately for up- and down-regulated genes, with only the top 20 results presented (Figures 3B,C; Supplementary Tables S3, S4). Genes highly expressed in LD were primarily enriched in key biological processes such as bone formation, while no direct enrichment related to IMF deposition was observed in the KEGG pathway analysis. Conversely, low-expressed genes in LD were mainly enriched in pathways associated with triglyceride metabolism, fatty acid oxidation, and lipid metabolism (Figures 3D,E). In the KEGG pathway analysis, these genes were predominantly enriched in signaling pathways such as PPAR signaling, steroid hormone biosynthesis, adipocyte metabolism, and arachidonic acid metabolism, which are closely linked to lipid and energy metabolism. The findings of this study are summarized in the following table.

Based on these results, we will focus on the transcriptional profiles of the BF high-IMF and low-IMF groups for subsequent research, aiming to identify differentially expressed lncRNAs, miRNAs, and mRNAs associated with IMF deposition, thereby providing critical insights into the underlying mechanisms of IMF deposition (Supplementary Tables S5, S6).

### 3.3 Differences in the expression profiles of mRNAs and ncRNAs in BF between high and low IMF conditions

**Figure 4:**
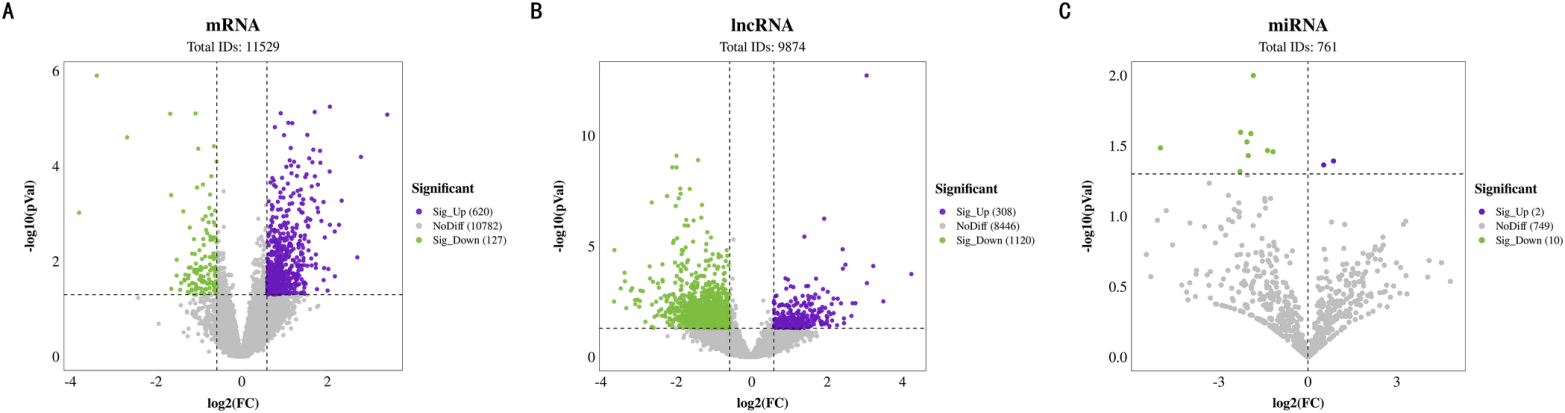
Differential expression identification of BF. **(A)** Volcano plot of differentially expressed mRNAs. **(B)** Volcano plot of differentially expressed lncRNAs. **(C)** Volcano plot of differentially expressed miRNAs.

In the transcriptome analysis of groups H and L, a total of 11,529 mRNAs were detected, among which 747 differentially expressed mRNAs (DE mRNAs) were identified. Specifically, 620 DE mRNAs were upregulated in group H, while 127 were downregulated (Figure 4A).

Regarding lncRNAs, a total of 9,874 were identified, including 330 known lncRNAs and 9,544 newly predicted ones. Differential expression analysis revealed that 1,428 lncRNAs were differentially expressed between the two groups, with 308 upregulated and 1,120 downregulated in group H (Figure 4B). These findings underscore the potential role of lncRNAs in regulating IMF content.

Additionally, the analysis of 761 miRNAs showed that 12 miRNAs were differentially expressed. Among them, miR-126a-3p_R-1 was upregulated in group H, whereas miR-874_R + 1, miR-1343-3p_R + 2, miR-210-3p, miR-1307-5p_R + 6, miR-3p-8435_305, miR-328, miR-99b, miR-370-3p_R-2, miR-128, miR-139-3p, and miR-139-3p were downregulated in group H (Figure 4C). The expression changes of these miRNAs may influence IMF accumulation by finely regulating gene expression networks.

**Figure 5:**
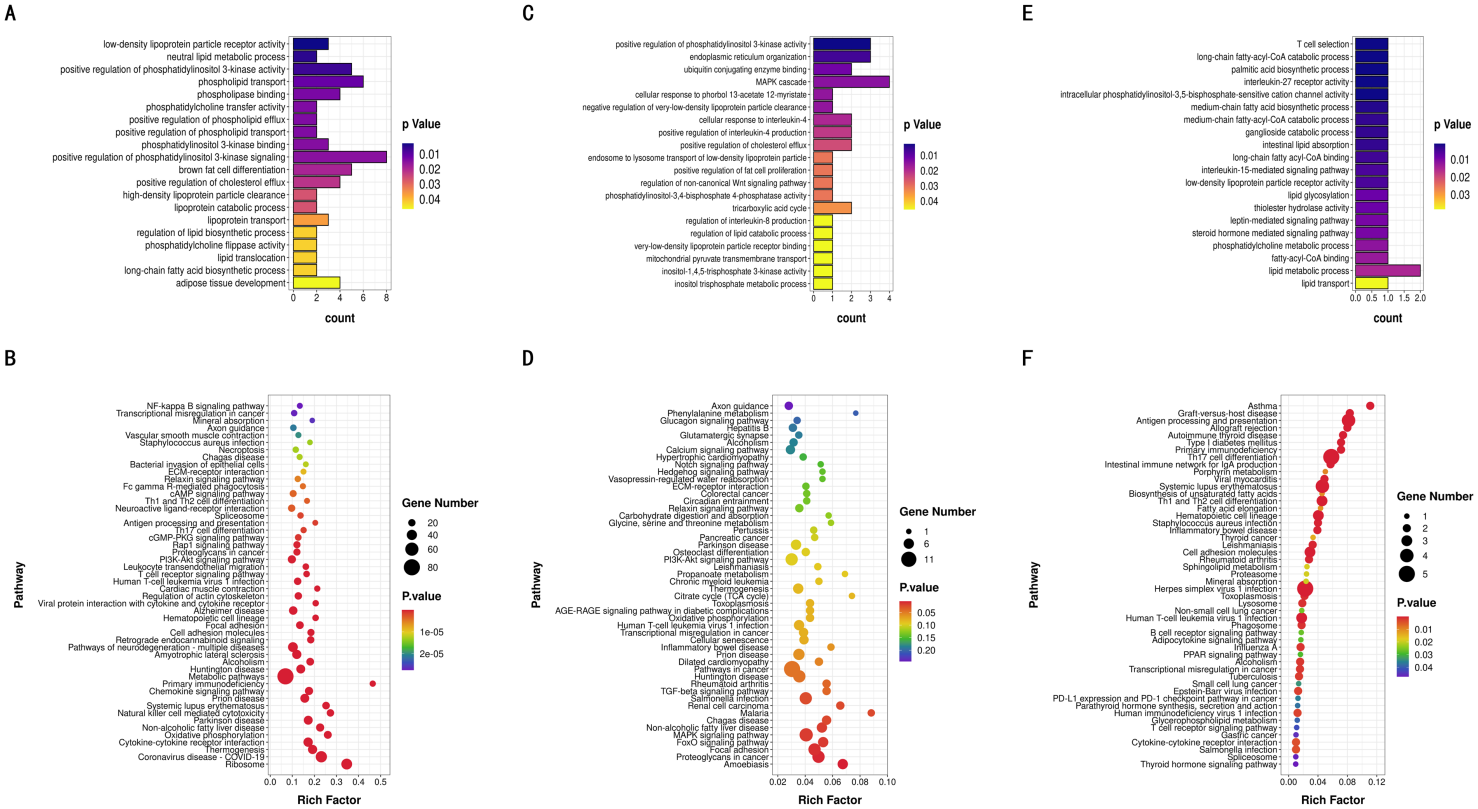
GO enrichment and KEGG pathway analyses. **(A)** GO analysis of the differentially expressed mRNAs. **(B)** KEGG pathway analysis of the differentially expressed mRNAs. **(C)** GO analysis of the differentially expressed lncRNAs. **(D)** KEGG pathway analysis of the differentially expressed lncRNAs. **(E)** GO analysis of the differentially expressed miRNAs. **(F)** KEGG pathway analysis of the differentially expressed miRNAs.

The differentially DE mRNAs identified between high and low IMF groups included LIPE, HADHA, LIPA, CPT1A, ADRB2, PLIN5, NR1D1, and other genes directly related to IMF deposition regulation. GO enrichment analysis of DE mRNAs indicated that these genes participated in 480 significantly enriched functional classes (*p <* 0.05), with 331 involved in Biological Process (BP), 73 in Cellular Component (CC), and 76 in Molecular Function (MF). Notably, DE mRNAs were significantly enriched in fatty acid production and lipid metabolism (*p <* 0.05), such as brown fat cell differentiation, regulation of lipid biosynthesis, long-chain fatty acid biosynthesis, and adipose tissue development (Figure 5A). KEGG enrichment analysis further revealed that these DE mRNAs are involved in pathways related to fat deposition metabolism, including Thermogenesis, Non-alcoholic Fatty Liver Disease, PI3K-Akt signaling pathway, and MAPK signaling pathway (*p <* 0.05) (Figure 5B).

From the 9,874 lncRNAs, a total of 1,169 target genes were obtained. Target genes of differentially expressed lncRNAs were enriched in GO functions, with 492 items significantly enriched (*p <* 0.05), many of which were related to lipid metabolism and the tricarboxylic acid cycle, including positive regulation of fat cell proliferation, tricarboxylic acid cycle, and regulation of lipid catabolic processes (Figure 5C). KEGG enrichment analysis showed that differentially expressed lncRNA target genes were significantly enriched in pathways such as the FoxO signaling pathway, MAPK signaling pathway, and Non-alcoholic Fatty Liver Disease (*p <* 0.05) (Figure 5D). In this study, 1,521 target genes of differentially expressed miRNAs (DE miRNAs) were identified. GO functional enrichment analysis of DE miRNA target genes revealed significant enrichment in triglyceride metabolism, fatty acid metabolism, adipocyte differentiation, and other related aspects (*p <* 0.05), including glycerol-3-phosphate catabolic process, fatty acid beta-oxidation, regulation of brown fat cell differentiation, and steroid metabolic processes (Figure 5E). DE miRNA target genes were also significantly enriched in 51 KEGG pathways, including the Glucagon signaling pathway and AMPK signaling pathway, both of which are related to lipid metabolism (Figure 5F). Through the synergistic action of these pathways, miRNAs play a complex regulatory role in intramuscular fat deposition.

### 3.4 Joint analysis of ncRNAs and mRNAs

To further understand the regulatory mechanisms of non-coding RNAs (ncRNAs) in different IMF deposition, we conducted a correlation analysis between the target genes of differentially expressed lncRNAs and mRNAs. This analysis aims to clarify the functions of lncRNAs in the regulation of fat deposition. The results section presents only the annotated bases, with a total of four mRNAs screened: one down-regulated (ST7) and three up-regulated (NR1D1, LIPE, and FCGR2B). Notably, NR1D1 and LIPE are associated with specific functions (Table 3). Additionally, Table 4 shows the results of the joint analysis of miRNAs and mRNAs. According to these results, the combination degree between miR-1343-3p_R + 2 and another miRNA was found to be high (Supplementary Table S7).

**Table 3:** Combined analysis of lncRNAs target genes and DE mRNAs.

LncRNAs ID	LncRNAs expression	Gene ID	mRNAs expression
MSTRG.30905.1	Down	*ST7* (Suppression of Tumorigenicity 7)	Down
MSTRG.7688.1	Up	*NR1D1* (Nuclear Receptor Subfamily 1 Group D Member 1)	Up
MSTRG.11866.1	Up	*LIPE* (Lipase E, Hormone Sensitive Type)	Up
MSTRG.36368.1	Up	*FCGR2B* (Fc Gamma Receptor IIb)	Up

**Table 4:** miRNAs target genes were analyzed in combination with DE mRNAs.

miRNAs ID	miRNAs expression	Gene ID	mRNAs expression
bta-miR-874_R + 1	Down	*CD4* (CD4 Molecule)	Up
bta-miR-1343-3p_R + 2	Down	*PSMB8* (Proteasome 20S Subunit Beta 8)	Up
oar-miR-370-3p_R-2	Down	*FCGR3A* (Fc Gamma Receptor IIIa)	Up
bta-miR-1343-3p_R + 2 bta-miR-874_R + 1	Down	*IFI30* (IFI30 Lysosomal Thiol Reductase)	Up
bta-miR-1343-3p_R + 2	Down	*DQA* (DQ Alpha 1)	Up
oar-miR-370-3p_R-2	Down	*CPT1A* (Carnitine Palmitoyltransferase 1A)	Up
bta-miR-1343-3p_R + 2 bta-miR-874_R + 1	Down	*HADHA* (Hydroxyacyl-CoA Dehydrogenase Trifunctional Multienzyme Complex Subunit Alpha)	Up
bta-miR-328	Down	*MMP14* (Matrix Metallopeptidase 14)	Up
bta-miR-874_R + 1 oar-miR-370-3p_R-2	Down	*CD53* (CD53 Molecule)	Up
bta-miR-328 bta-miR-874_R + 1	Down	*ATP1A1* (ATPase Na+/K+ Transporting Subunit Alpha 1)	Up

### 3.5 Construction of lncRNA-miRNA-mRNA network related to intramuscular fat deposition

**Figure 6:**
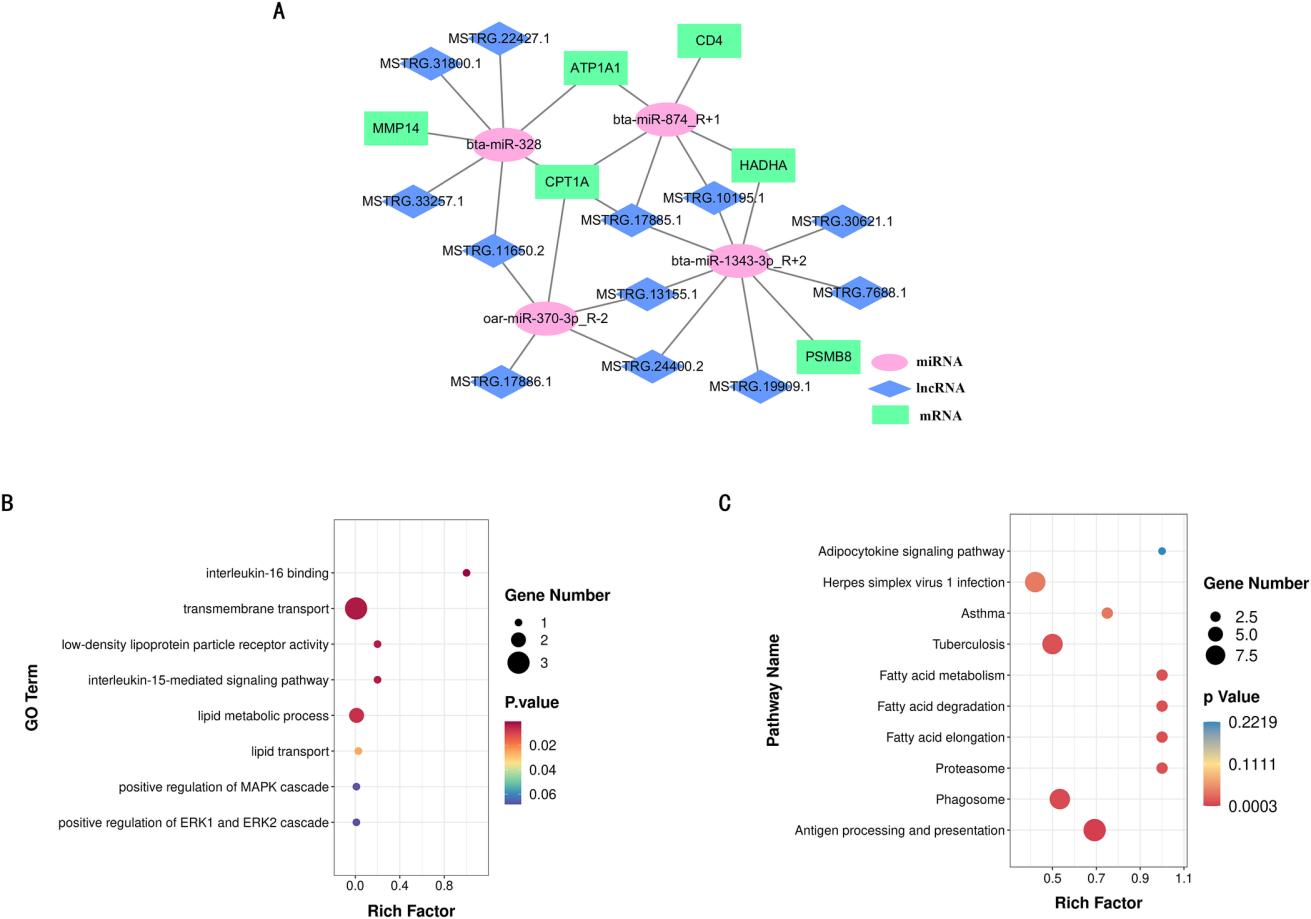
LncRNA-miRNA-mRNA co-expression network. **(A)** lncRNA-miRNA-mRNA co-expression network. **(B)** GO function rich distribution map of key genes. **(C)** KEGG function rich distribution map of key genes.

To further identify the key competing endogenous RNA (ceRNA) target relationships that regulate IMF deposition in the biceps femoris muscle of Ujumqin sheep, we integrated the results from our previous association analyses to construct a comprehensive ceRNA network encompassing lncRNAs, miRNAs, and mRNAs. In this network, target relationships were required to meet stringent criteria: a TargetScan score of at least 90 and a miranda Energy less than −20 (Figure 6A). The network comprised 25 nodes, including 12 lncRNAs, 4 miRNAs, and 6 mRNAs, illustrating the intricate ceRNA regulatory interactions. The network’s complexity is highlighted by the fact that a single miRNA can target multiple lncRNAs and mRNAs, and different miRNAs can target the same lncRNA or mRNA. This diversity in lncRNA-miRNA-mRNA interactions leads to a variety of biological functions, positioning these non-coding RNAs as promising candidates for subsequent functional analyses. Furthermore, we evaluated the functions of the central target genes within this ceRNA network using Gene GO and KEGG analyses. The results showed significant enrichment in lipid metabolism and lipid transport functions within the GO categories. Additionally, these key target genes were significantly enriched in fatty acid metabolism and fat cell signaling pathways according to KEGG analyses (Figures 6B,C).

### 3.6 HADHA is the target gene of bta-miR-1343-3p_R + 2, and lncRNA-MSTRG.13155.1 releases HADHA through ceRNA

We aimed to verify the targeting relationship within the lncRNA-miRNA-mRNA regulatory network using the dual luciferase reporter assay, focusing on the interaction between MSTRG.13155.1, bta-miR-1343-3p_R + 2, and HADHA.

**Figure 7:**
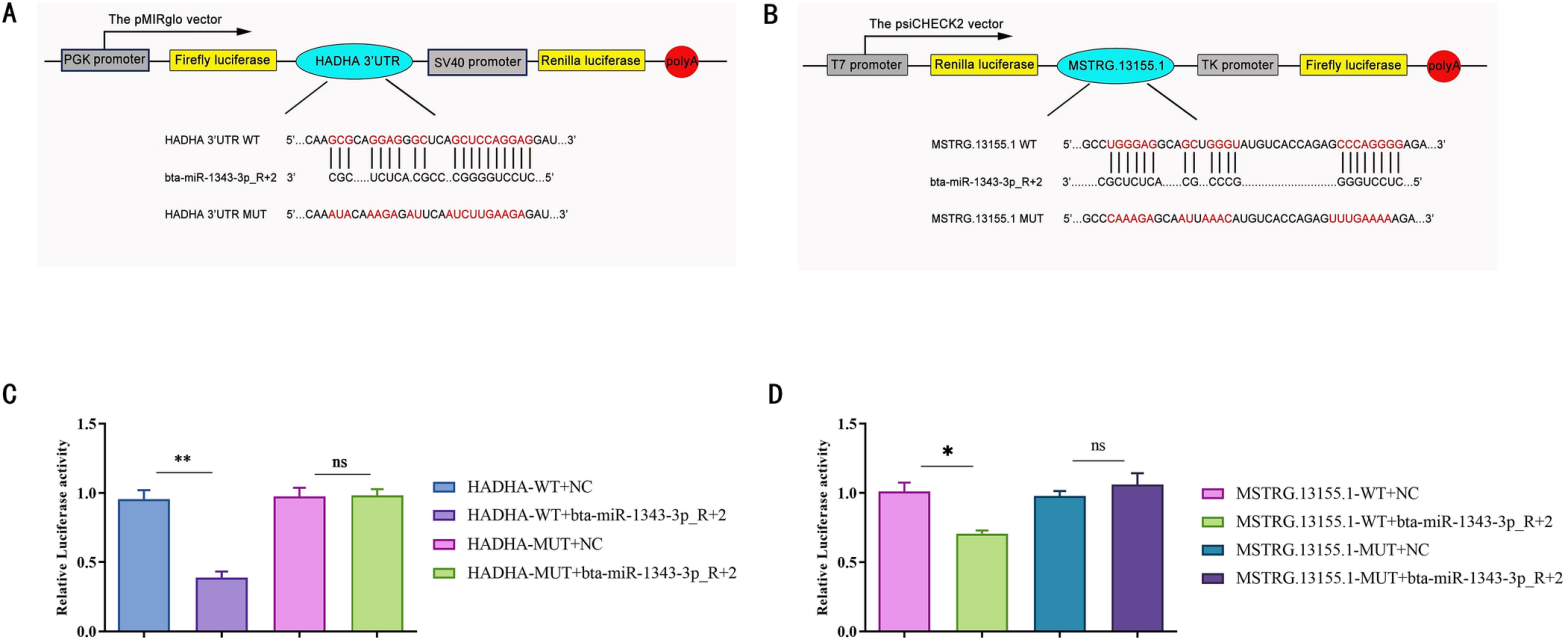
Verification of miR-1343-3p_R + 2 target binding for HADHA 3’UTR while MSTRG.13155.1 released the expression of HADHA by competing endogenous RNAs. **(A)** Prediction of binding sites of miR-1343-3p_R + 2 and HADHA in RNAhybrid. **(B)** Prediction of binding sites of miR-1343-3p_R + 2 and MSTRG.13155.1 in RNAhybrid. **(C)** Luciferase assay in 293-t cells co-transfected with pMirGLO-HADHA-3 ‘-UTR-WT/MUT and miR-1343-3p_R + 2 mimics (**p < 0.001, *p < 0.05). **(D)** Luciferase assay in 293-t cells co-transfected with pMirGLO-MSTRG.13155.1-3 ‘-UTR-WT/MUT and miR-1343-3p_R + 2 mimics (**p < 0.001, *p < 0.05).

We hypothesized that their binding site exhibits a high binding affinity. To test this, we co-transfected HEK-293 T cells with constructs containing the potential target gene 3´UTR or sequences with mutated seed regions alongside bta-miR-1343-3p_R + 2 mimics (Figures 7A,B). The results supported our hypothesis: bta-miR-1343-3p_R + 2 mimics significantly reduced the luciferase activity of HADHA-WT-3´UTR (*p <* 0.01), with no significant inhibition observed with HADHA-MUT3´UTR compared to the negative control group (Figure 7C). These findings indicated that the mutation at the binding site disrupted the interaction between bta-miR-1343-3p_R + 2 and HADHA, thereby confirming the specific targeting effect of bta-miR-1343-3p_R + 2 on HADHA. Similarly, bta-miR-1343-3p_R + 2 mimics significantly decreased the luciferase activity of MSTRG.13155.1-WT-3´UTR (*p <* 0.05), while no significant inhibition was detected with MSTRG.13155.1-MUT3´UTR compared to the negative control group (Figure 7D).

These findings lead us to conclude that HADHA is a target gene of bta-miR-1343-3p_R + 2 and that the lncRNA MSTRG.13155.1 functions as a ceRNA to regulate HADHA expression. These findings indicated that the mutation at the specific site inhibited the binding of bta-miR-1343-3p_R + 2 to MSTRG.13155.1, thereby confirming this site as the binding site for bta-miR-1343-3p_R + 2. Consequently, it can be inferred that HADHA is a potential target gene of miR-1343-3p_R + 2, and lncRNA-MSTRG.13155.1 may release HADHA via the ceRNA mechanism.

### 3.7 RT-qPCR verification

**Figure 8:**
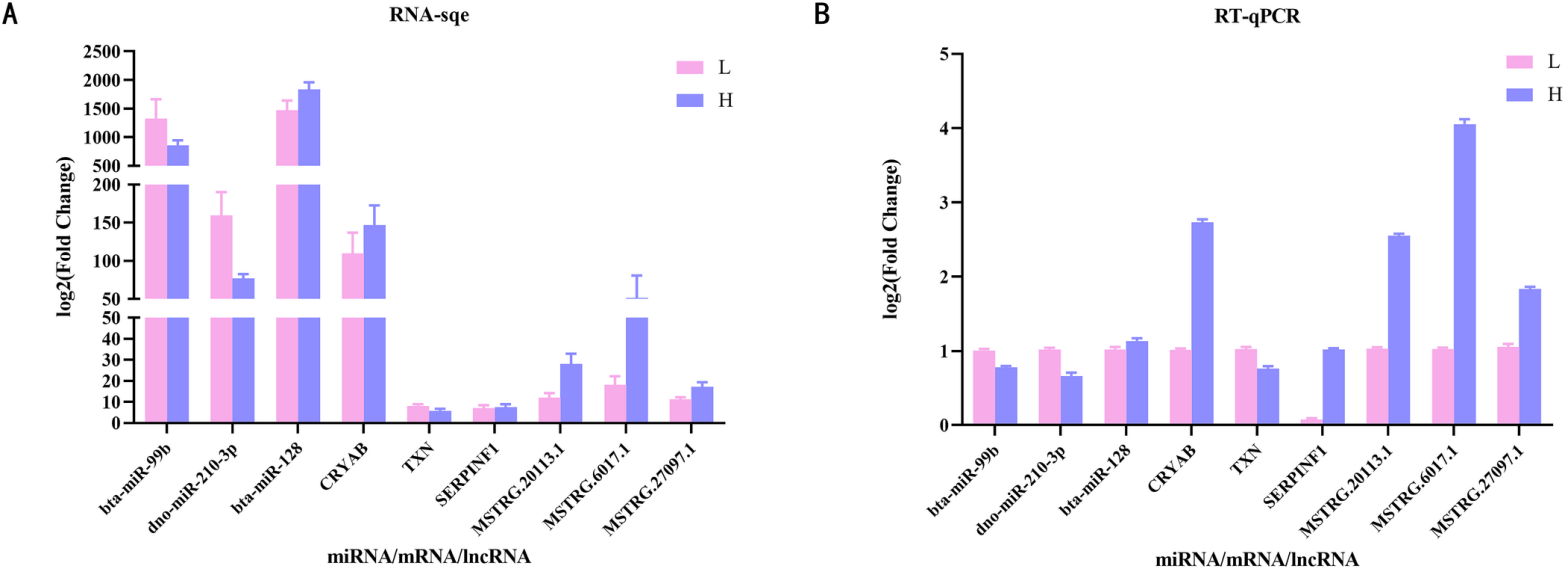
Validation of differentially expressed mRNAs, differentially expressed miRNAs and differentially expressed lncRNAs through RT-qPCR. **(A)** Expression levels of genes by RNA-Seq. **(B)** RT-qPCR verification results of 3 mRNAs, 3 miRNAs and 3 lncRNAs. RT-qPCR, Reverse transcriptase quantitative real-time PCR.

We randomly selected three DE miRNAs (bta-mir-99b, dno-mir-210-3p and bta-mir-128), three DEMs (CRYAB, SERPINF1 and TXN) and three DE lncRNAs (MSTRG.20113.1, MSTRG.6017.1 and MSTRG.27097.1) were subjected to RT-qPCR, and the results were shown in Figure 8. The expression trends of the genes were consistent with the changes in the expression trends in the sequencing results, indicating that the sequencing results were reliable for subsequent analysis.

## 4 Discussion

The mutton sheep industry has emerged rapidly in China as a promising sector, playing a crucial role in the development of the livestock industry. The demand for mutton has shifted from quantity to quality. Previous studies (23–25) have demonstrated that the Ujumqin lamb breed outperforms other breeds in terms of slaughter rate and carcass weight. Ujumqin sheep, an excellent indigenous breed in Inner Mongolia, exhibit strong disease resistance and adaptability to natural grazing conditions. They are renowned for their rapid growth rate, high meat yield, superior meat quality, and efficient fat deposition. In livestock production, skeletal muscle growth and development directly influence mutton yield, while IMF deposition significantly affects tenderness, flavor, and juiciness (26). To enhance the mutton quality of Ujumqin sheep, we investigated the IMF deposition mechanism, which is regulated by complex mechanisms at both transcriptional and post-transcriptional levels (27, 28). Fatty acids, as key lipid components, substantially impact meat product quality (29). This study identified 31 fatty acids in Ujumqin mutton, with consistent fatty acid composition across different parts. Among saturated fatty acids, palmitic acid and arachidonic acid were found in higher concentrations. Oleic acid was the most abundant monounsaturated fatty acid, while polyunsaturated fatty acids primarily consisted of trans-linoleic acid and *α*-linolenic acid. Notably, the LD group exhibited significantly higher levels of saturated fatty acids compared to the BF group (*p* < 0.05), whereas the BF group had significantly higher levels of unsaturated fatty acids (*p* < 0.05). Oleic acid, known for its ability to reduce blood cholesterol, and α-linolenic acid, an n-3 series fatty acid that generates DHA (docosahexaenoic acid) and EPA (eicosapentaenoic acid) with physiological benefits, were particularly prominent. DHA supports health maintenance and development, while EPA reduces blood lipids and cholesterol and improves brain nerve function (30). Therefore, the fatty acid profile of the BF group is characterized by a higher content of unsaturated fatty acids, especially oleic acid and α-linolenic acid, distinguishing it from the LD group. Additionally, the composition of fatty acids is associated with the fiber type composition of different muscle regions. Research has shown that muscle fibers can be categorized into slow-twitch oxidative fibers (Type I), fast-twitch oxidative fibers (Type IIA), and fast-twitch glycolytic fibers (Type IIB) based on their contraction speed and metabolic characteristics (31, 32). The Ujumqin sheep is a grazing breed, and year-round grazing results in a higher proportion of fast and slow muscle fibers (such as Type IIA and Type IIX) in the biceps femoris muscle (33). These fiber types are more suited for rapid energy metabolism, in which unsaturated fatty acids are more readily utilized and stored.

By comparing the differentially expressed genes between LD and BF, we found that genes in LD were primarily enriched in key biological functions such as bone formation, whereas genes in BF were mainly enriched in pathways related to triglyceride metabolism, fatty acid oxidation, and lipid metabolism. KEGG pathway analysis further confirmed the involvement of BF in IMF deposition, with pathways such as PPAR signaling, steroid hormone biosynthesis, adipocyte metabolism, and arachidonic acid metabolism being closely related to lipid and energy metabolism. In the aforementioned study, we found that the content of unsaturated fatty acids in BF was relatively high. Fatty acids are the primary components of fat and play a crucial role in determining meat quality (34). Among them, PUFAs are particularly important as they contribute significantly to the flavor profile of meat. Based on these findings, we selected the transcriptional profiles of the IMF group with high BF and the IMF group with low BF as the foundation for subsequent research.

Drawing upon the ceRNA hypothesis, lncRNAs can function as miRNA sponges, thereby facilitating crosstalk with mRNAs (35). This mechanism offers a potential avenue through which lncRNAs may regulate IMF deposition in meat sheep, warranting further investigation. Studies have demonstrated that lncRNAs play a pivotal role in fat deposition. A single lncRNA can harbor multiple binding sites for a diverse array of miRNAs, thereby regulating numerous target mRNAs and contributing to a dynamic and intricate regulatory network. This complexity enhances the sophistication of lncRNA regulatory mechanisms. For instance, IMFNCR functions as a ceRNA, sequestering miR-128-3p and miR-27b-3p, which results in elevated PPARG expression and subsequently promotes intramyocellular adipocyte differentiation (36). The research discovered that lncRNA NEAT1, through its interaction with miR-146a-5p, targets ROCK-1 (RHO-associated coiled-coil containing protein kinase 1) and further modulates the AMPK/SREBP pathway, ultimately facilitating fat deposition (37). To delve into the regulatory relationships among lncRNAs, miRNAs, and mRNAs, we initially employed prediction tools available on public websites to identify the targets of differentially expressed miRNAs (DE miRNAs) and differentially expressed lncRNAs (DE lncRNAs). Based on these predictions, we constructed the lncRNA-miRNA-mRNA interaction network using Cytoscape software, which visually illustrates the regulatory interactions among these three types of molecules. Subsequently, we validated the expression levels of selected mRNAs, lncRNAs, and miRNAs within the network using RT-qPCR technology. The validation results were consistent with the sequencing data, further confirming the reliability of our sequencing results.

In this study, we identified key regulatory factors involved in the regulation of intramuscular fat deposition in sheep, including mRNAs, lncRNAs, and miRNAs, and constructed a regulatory network based on this information. Notably, genes related to lipid metabolism and fatty acid oxidation, such as HADHA and CPT1A, are present within this network. HADHA (hydroxyacyl-CoA dehydrogenase trifunctional multienzyme complex subunit alpha) is a mitochondrial enzyme that regulates the biosynthesis of carnitine, an essential endogenous metabolite required for the transport of fatty acids into mitochondria for *β*-oxidation (38). Our findings indicate that HADHA may exert a significant influence on IMF deposition through the ceRNA regulatory network. Furthermore, CPT1A encodes carnitine palmitoyltransferase-1, a key mitochondrial enzyme involved in the β-oxidation of long-chain fatty acids. In our study, CPT1A was upregulated in sheep with higher IMF content, which aligns with previous reports demonstrating a positive correlation between CPT1A expression levels and IMF content (39). In addition to the aforementioned genes, our analysis revealed that other genes within the network are also implicated in adipogenesis, either directly or indirectly. Huang et al. (40) utilized a lentiviral delivery system to express the mouse ATP1A1 transgene in 3 T3-L1 adipocytes and identified that the conserved N-terminal vesicle-binding motif of ATP1A1 is a critical mediator and potential therapeutic target for lipodystrophy syndrome. Furthermore, the infiltration of CD4 T cells in adipose tissue has been shown to alter the local microenvironment through the secretion of cytokines such as TNF-*α* and IL-6, which can inhibit adipocyte differentiation or promote lipolysis (41). Arimochi et al. (42) demonstrated that Psmb8, a catalytic subunit of the immunoproteasome, directly regulates the differentiation of preadipocytes and influences their maturation into adipocytes. Additionally, Kopinke et al. (43) revealed that MMP-14 can promote adipogenesis in 3 T3-L1 preadipocytes even *in vitro*, suggesting that MMP-14 can directly affect cellular differentiation. Collectively, these findings underscore the relevance of the differentially expressed genes identified in our study, highlighting their potential to reflect the impact on IMF deposition. The results of GO and KEEG enrichment analyses further confirm the above view. In the GO annotation, these DEMs mainly function in biological processes that are closely related to lipid formation and deposition, such as neutral lipid metabolic process, brown fat cell differentiation, regulation of lipid biosynthetic process, long-chain fatty acid biosynthetic process and adipose tissue development. In addition, we have identified a number of genes in processes such as the immune system, signaling and cell proliferation and differentiation, and studies have shown that signaling between adipocytes and immune cells can greatly influence adipose tissue function (44). KEGG enrichment analysis revealed that the target genes Non-alcoholic fatty liver disease, PI3K-Akt signaling pathway, FOXO signaling pathway, MAPK signaling pathway and Phospholipase D signaling pathway, etc., which are essential in lipid deposition. Both mRNAs and lncRNAs are significantly enriched in these pathways except for the PI3K-Akt signaling pathway, which is associated with the formation of long-chain polyunsaturated fatty acids in sheep and has been shown to be a negative regulator (45). The above studies suggest their potential regulatory role on IMF deposition in sheep. However, there are still many DEMs with unknown functions, and whether they are related to lipid deposition still needs further research.

Notably, non-coding RNAs (ncRNAs) exert significant effects on lipid formation and metabolism (46, 47). However, it is crucial to acknowledge that the functionality of lncRNAs is not conserved across species. Consequently, previous research conducted in humans and mice has limited applicability as a reference in the context of livestock production (48). Furthermore, the majority of studies have concentrated on perirenal and subcutaneous fat, with intramuscular fat receiving comparatively scant attention. The ontogeny and development of IMF diverge from other adipose tissues, which hinders the direct extrapolation of findings from other adipose tissues to IMF tissues (49, 50). In our study, we observed that the majority of lncRNAs displayed a moderate-to-low expression profile. This suggests that these lncRNAs may influence the differentiation process of preadipocytes by interacting with related genes during cell differentiation. In order to further investigate the mechanism of lncRNA action, based on KEGG and GO enrichment analyses as well as the results of differentially expressed genes, we selected 12 important lncRNAs that might be associated with IMF deposition and predicted their target miRNAs. These results provide a theoretical basis for investigating the regulatory relationship between lncRNAs and IMF deposition in sheep. In our study, MSTRG.24400.2, MSTRG.17885.1, MSTRG.13155.1, MSTRG.10195.1 and their target genes were identified in phospholipid translocation, cholesterol metabolic process, lipoprotein biosynthetic process and other pathways associated with fat deposition, and all target the network core gene bta-miR-1343-3p. MiR-1343-3p was found to activate the PI3K signaling pathway through lncRNA PI3K signaling pathway (51) and was also identified to inhibit fatty acid metabolic processes (52). The above results support our findings in these studies to some extent. Therefore, the lncRNAs we identified in this study may have potential applications as biomarkers in the regulation of intramuscular fat deposition. Looking ahead, the MSTRG.13155.1-bta-miR-1343-3p_R + 2 axis is poised to emerge as a significant candidate pathway for elucidating the mechanisms underlying IMF deposition in sheep.

MicroRNAs a single-stranded non-coding RNAs of 18–25 nucleotides in length, can directly target mRNAs to regulate gene expression and often interact with mRNAs and lncRNAs through the ceRNA network to play regulatory roles (53). In this study, a total of 761 miRNAs were obtained by sequencing, of which 11 DE miRNAs were identified between groups H and L. Through functional enrichment analysis of their target genes, we identified a number of miRNAs, including miR-370-3P (54) and miR-328 (55) were found to be key genes regulating adipogenesis, which is consistent with previous findings. Other well-documented examples of ceRNA regulatory mechanisms include lncRNA IMFNCR, which acts as a ceRNA to sequester miR-128-3p and miR-27b-3p. This interaction leads to elevated PPARG expression and subsequently promotes the differentiation of intramyocellular adipocytes (36). Additionally, Chen et al. (37) revealed that lncRNA NEAT1 targets ROCK-1 (Rho-associated coiled-coil containing protein kinase 1) via miR-146a-5p, thereby influencing the AMPK/SREBP pathway and facilitating fat deposition. In our dual-luciferase reporter assays, we observed that high expression of bta-miR-1343-3p_R + 2 significantly inhibits HADHA expression. However, there is currently no direct evidence to suggest that MSTRG.13155.1 can competitively bind to bta-miR-1343-3p_R + 2 via a ceRNA mechanism to relieve the inhibition of HADHA expression. Therefore, the precise interaction between MSTRG.13155.1 and bta-miR-1343-3p_R + 2 requires further investigation through competitive binding assays and additional experimental approaches.

These results indicate that the genes we have identified are likely to play significant roles in regulating IMF deposition in sheep. Moving forward, we plan to delve deeper into the specific mechanisms of action of these candidate mRNAs and non-coding RNAs, thereby providing a clearer understanding of their roles in IMF deposition in sheep.

## 5 Conclusion

In this study, we performed whole-transcriptome sequencing of high-IMF and low-IMF group samples from the biceps femoris muscle of Ujumqin sheep, identifying differentially expressed mRNAs, miRNAs, and lncRNAs. By constructing a ceRNA network, we systematically analyzed the lncRNA-associated regulatory mechanisms involved in IMF deposition in the biceps femoris muscle of Ujumqin sheep for the first time. We further validated the interaction between MSTRG.13155.1, bta-miR-1343-3p_R + 2, and HADHA in this network using a dual-luciferase reporter assay. These findings reveal potential regulatory factors and molecules associated with IMF deposition in Ujumqin sheep, providing a significant theoretical basis for further investigation into the regulatory mechanisms underlying IMF deposition.

## Data Availability

This study includes two databases. The miRNA database is available at http://www.ncbi.nlm.nih.gov/bioproject/1232690 (BioProject accession number: PRJNA1234690). The lncRNA database can be accessed at https://www.ncbi.nlm.nih.gov/bioproject/PRJNA1217802 (BioProject accession number: PRJNA1217802). These data are publicly available and can be freely accessed and used by any researcher.

## Footnotes

^1^ http://www.Bioinformatics.babraham.ac.uk/projects/fastqc/

^2^ http://cpc2.cbi.pku.edu.cn

^3^ https://github.com/www-bioinfo-org/CNCI

^4^ https://mirdb.org/
